# Model for prompt and effective classification of motion recovery after stroke considering muscle strength and coordination factors

**DOI:** 10.1186/s12984-019-0611-z

**Published:** 2019-11-04

**Authors:** Álvaro Costa-García, Ken-ichi Ozaki, Hiroshi Yamasaki, Matti Itkonen, Fady Alnajjar S., Shotaro Okajima, Masanori Tanimoto, Izumi Kondo, Shingo Shimoda

**Affiliations:** 1 Intelligent Behaviour Control Unit, RIKEN Center of Brain Science, CBS-Toyota Collaboration Center in the Nagoya Science Park Research and Development Center, 2271-130 Anagahora, Shimoshidami, Moriyama-ku, Aichi-ken, Nagoya, 463-0003 Japan; 2 National Center for Geriatrics and Gerontology, 7-430, Morioka cho, Aichi-ken, Ohbu, 474-8511 Japan; 3 Intelligent Robot Interaction Lab, College of Information Technology, United Arab Emirates University, Abu Dhabi, 15551 United Arab Emirates

**Keywords:** Motion performance, Stroke recovery, Electromyography, Muscle synergies, Muscle mirror symmetry, Muscle effective strength, Motor strength gain

## Abstract

**Background:**

Muscle synergies are now widely discussed as a method for evaluating the existence of redundant neural networks that can be activated to enhance stroke rehabilitation. However, this approach was initially conceived to study muscle coordination during learned motions in healthy individuals. After brain damage, there are several neural adaptations that contribute to the recovery of motor strength, with muscle coordination being one of them. In this study, a model is proposed that assesses motion based on surface electromyography (sEMG) according to two main factors closely related to the neural adaptations underlying motor recovery: (1) the correct coordination of the muscles involved in a particular motion and (2) the ability to tune the effective strength of each muscle through muscle fiber contractions. These two factors are hypothesized to be affected differently by brain damage. Therefore, their independent evaluation will play an important role in understanding the origin of stroke-related motor impairments.

**Results:**

The model proposed was validated by analyzing sEMG data from 18 stroke patients with different paralysis levels and 30 healthy subjects. While the factors necessary to describe motion were stable across heathy subjects, there was an increasing disassociation for stroke patients with severe motor impairment.

**Conclusions:**

The clear dissociation between the coordination of muscles and the tuning of their strength demonstrates the importance of evaluating these factors in order to choose appropriate rehabilitation therapies. The model described in this research provides an efficient approach to promptly evaluate these factors through the use of two intuitive indexes.

## Background

As a consequence of the population aging around the world, stroke has become a widespread concern [1]. Depending on the brain area and size of the injury, the consequences of motor impairments vary significantly [2]. It has been widely shown that in order to enhance stroke rehabilitation with respect to effective motor recovery, it is necessary to start therapy shortly after the cerebrovascular accident [3–5]. Therefore, the use of inefficient treatments during early stages of rehabilitation, might result in an insufficient motor recovery. More than 60% of stroke survivors have remaining motor paralysis, resulting in serious social cost and affecting their quality of life for the rest of their lives [6, 7].

In recent years, the concept of muscle synergies has been widely discussed to clarify the biological basis for activating the redundant musculoskeletal system of stroke survivors [8–10]. According to this model, during motor learning, the plastic nature of neurons creates modules or neural networks (called synergies) that are specialized for different tasks [10, 11]. Synergies control the contraction of a set of muscles using a low-dimensional set of control commands originating from the brain [12–15]. It has been proposed that use of the remaining neural pathways could potentiate post-stroke recovery. A study on stroke patients with affected frontal motor cortical areas shows similar synergies in both arms (i.e., paretic and non-paretic) irrespective of their motion performance [16], suggesting a synergetic behavior independent of task constraints. In addition, another study [17] reveals the natural emergence of new muscle synergies during the learning process associated to the control of a myoelectric interface. However, these findings conflicts with other studies that show abnormal synergies appearing for the paretic arm of stroke patients [9, 18, 19]. This phenomenon was further evaluated by [20] in 31 stroke survivors. Paretic and non-paretic upper limb synergies were compared, showing three different behaviors on the non-paretic side: preservation, merging, and fractionation of synergies. Each behavior was affected differently by the level of motor impairment and poststroke duration. These results predict a wide range of stroke conditions from the perspective of muscle synergies, highlighting the importance of individual patient-by-patient evaluations. In order to clarify the origin of these results, it is necessary to understand the reasons behind the development of the muscle synergy approach and contrast them with the main concerns in the field of stroke rehabilitation.

Learned motions are perceived by healthy individuals as simple and easy, even though they require the complex coordination of muscles. The concept of muscle synergies was originally developed to explain the neural processes that provide the brain with tools to reduce the kinesiological complexity behind this coordination. However, after a stroke, the failure in motor control occurs not in the spinal cord but in the brain. Although studying the appearance of new muscle synergies after brain damage is necessary, current knowledge on this topic is not enough to choose an appropriate rehabilitation treatment. Neural recovery starts at the brain level, and depending on the severity of the injury will trigger changes at the spinal and muscular levels [21]. In this scenario, motor recovery relies not only on the neural process of motor coordination but also on all the physiological processes underlying motor strength gaining [22]. It is currently agreed that gains in voluntarily motor strength depend on two main factors: neural adaptations and muscle hypertrophy [22]. Brain damage alone does not affect muscle fiber condition; therefore, the loss of motor strength directly after a stroke is related to malfunctions of neural adaptations. The current literature enumerates these issues as malfuncitons of potentiation of neural connectivity, motor unit synchronization, muscle coordination, and learning [23]. The study of these neural adaptations during motion recovery is a challenging task due to the complexity of the neural system and current technological limitations on their measurement. However, it is possible to infer their effects on surface electromyography (sEMG) signals by evaluating muscle activation according to the following two factors. (1) The neural adaptations in charge of muscle coordination and learning affect the distribution of electrical power among all muscles contributing to the motion. (2) Structural and synaptic neural connectivity and the processes underlying the recruitment of motor units affect the ability to tune the effective strength of each muscle contraction. Assuming the symmetrical properties of the healthy human body, a in this study a model is proposed for evaluating these two factors during periodic symmetrical motions through the analysis of sEMG signals.

During motion in healthy individuals, the mechanisms of muscle coordination and the tuning of effective muscle strength compensate for each other, thereby creating stable muscle synergies. In this research, it is hypothesized that after brain damage, these two factors are affected differently (i.e., disassociated), thus generating a wider range of motor impairments. As these factors are directly related to the neural adaptations underlying motor recovery, their measurement will facilitate the prompt identification of the neural origins of the motor impairment, and therefore the selection of an effective rehabilitation therapy.

## Materials and methods

### Motions and experimental procedure

**Fig. 1:**
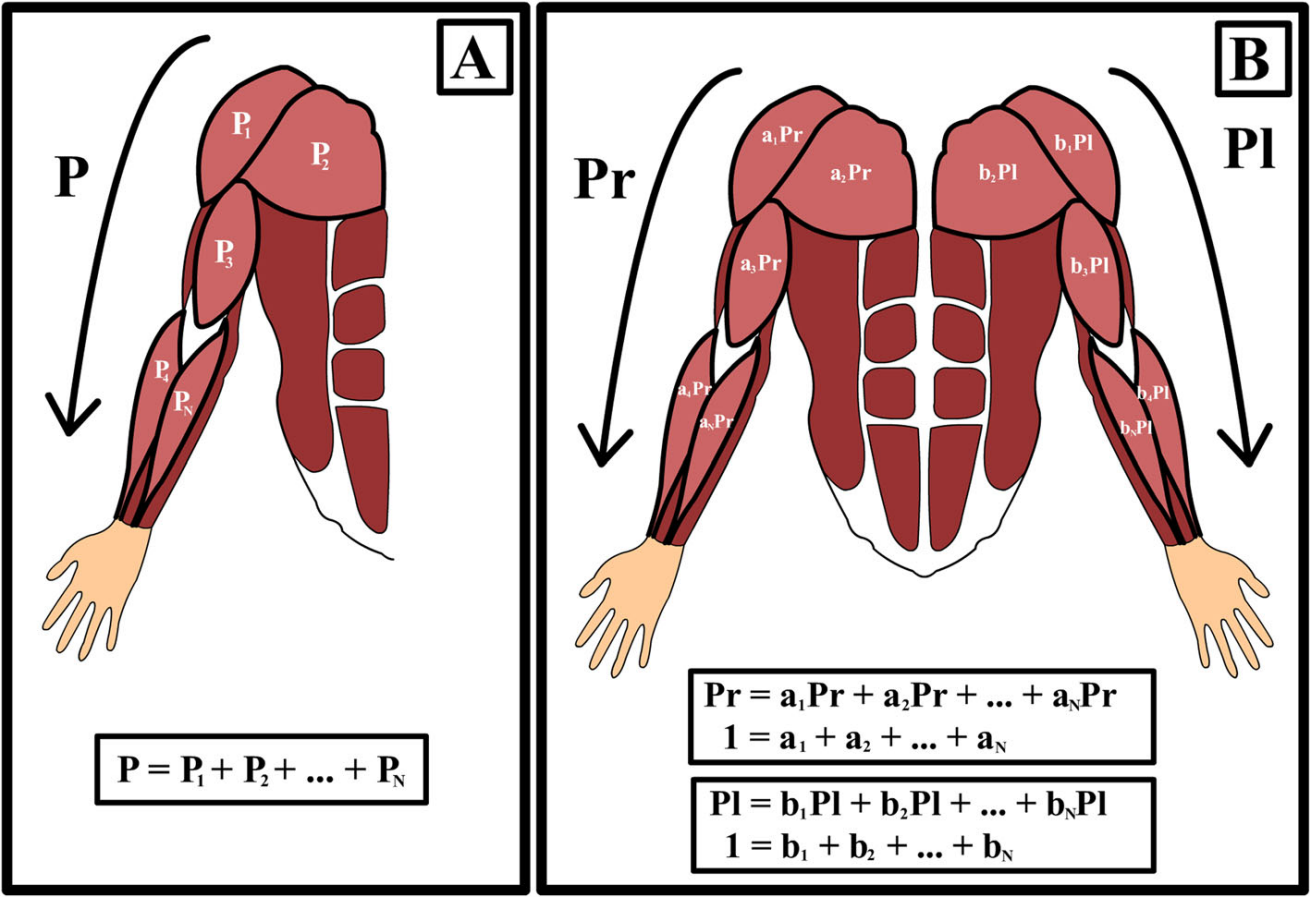
Graphical representation of the model. **a** Basic representation of the proposed model, in which the total electrical power needed to perform the motions (*P*) is represented as the summation of the individual electrical powers applied for each contributing muscle (*a*_*i*_*P*). **b** Model applied to bimanual motions

**Fig. 2:**
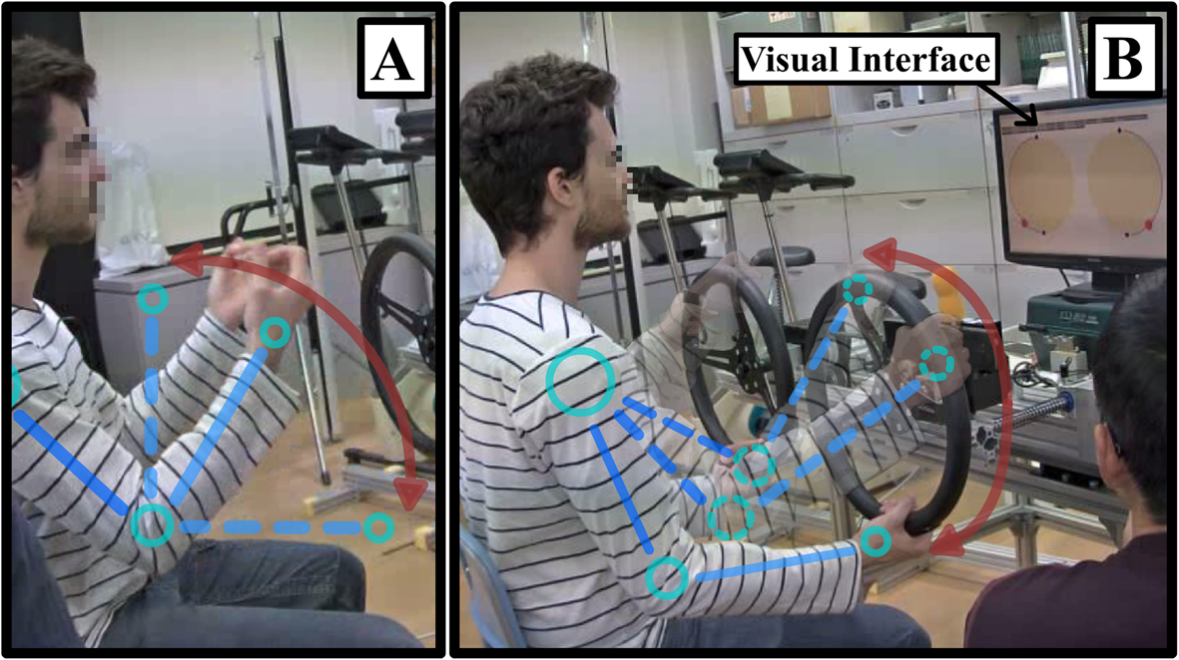
Motions. **a** For elbow flexion and extension, subjects flexed and extended both arms at an angle of 90 degrees as shown in the image. **b** For steering cycles, the subjects moved their hands as guided by the steering wheel system following the trajectory represented in the image. For both motions, a visual interface showing circles moving up and down was used to synchronize the motion of users at the desired speed (0.25 Hz)

Subjects were asked to perform two different bimanual symmetric motions which have been proved not only efficient tasks to asses stroke [24, 25], but also as a promising tool for upper-limb motor recovery [26–29]. Both motions (illustrated in Fig. 1) were chosen to highlight different patterns of muscle activation. The first motion consisted of a repeated elbow flexion of approximately 90 degrees between the arm and forearm (Fig. 1a). The second motion was supported by a dual steering system and consisted of motions along half steering cycles from the lower to upper position of the wheels, as shown in Fig. 2b. Subjects sat on a chair while performing both motions. A visual interface consisting of a red circle oscillating at a constant frequency was used to synchronize participants’ motion speed with the desired frequency for the experiment (0.25 Hz, 1 cycle in 4 s). The threshold to set the motion speed was chosen ad hoc based on previous experience of the authors to fit the physical conditions of severe stroke patients so all participants were able to perform the experiment.

The experimental trial consisted of 20 s of motion followed by 20 s of rest. The experimental session was composed of three 20-s elbow flexion trials and three 20-s dual steering trials. These trials were presented in a randomized order to reduce possible biases in subjects’ motor behavior owing to constant repetition of the same task.

### Participants

EMG data were recorded from 18 stroke patients (9 men and 9 women) between the ages of 66 and 96 years (74.88 ±9.63). Table 1 summarizes information about the patients’ paretic side and paralysis level according to the Stroke Impairment Assessment Set (SIAS) as measured on their paretic arm [30]. EMG data was recorded from patients on different session performed in a 6 months time period. During this time, patients were submitted to daily rehabilitation therapies which modify their motion. Data presented on this work belong to stroke patients that did not show changes in their SIAS level during the experimental period.In addition, patient data presented several analysis challenges associated with the availability of patients and their capability of performing the two tasks correctly. Accordingly, Table 1 also shows the number of trials obtained for each patient and motion. During experiments with patients there were several factors affecting the quality of the sEMG signals such as bad posture or electrodes touching with the clothes and disconnecting. During measurements sEMG signals were visualized by the experimenter and trial showing low signal quality were manually discarded from analysis. In addition, EMG data from 30 healthy right-handed subjects were also recorded. To facilitate validation, healthy subjects were divided into two groups depending on their age. The first group (healthy young) included 10 subjects (6 men and 4 women) aged between 25 and 44 years (34.88 ±7.47). The second group (healthy elder) included 20 subjects (7 men and 13 women) aged between 45 and 87 years (69.63 ±10.96). For healthy subjects, it was possible to increase the number of trials to compensate for the appearance of equipment- and protocol-induced noise in the sEMG signals. Each healthy elder subject performed three trials of each motion (20 *healthy* *elder* × 3 *trials* = 60 *trials* per motion). Finally, each healthy young subject was asked to perform six trials per motion (10 *healthy* *young* × 6 *trials* = 60 *trials* per motion). Healthy subjects did not present any known diseases and had no medical record history associated with motor disfunction. All subjects were previously informed about the experimental procedure and provided written informed consent, in accordance with the Declaration of Helsinki. The experimental procedure was approved by the ethics committees of the National Center for Geriatrics and Gerontology and RIKEN.

**Table 1:**
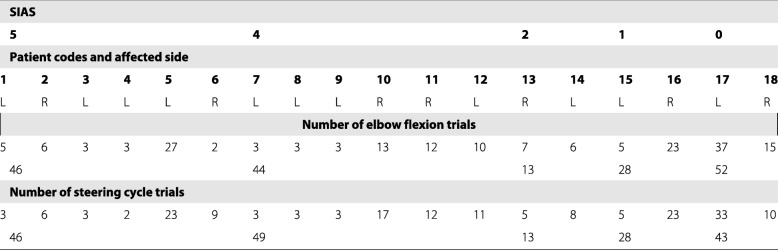
Patient summary

Stroke patients participating in the experiment were grouped according to their paretic arm motion performance as measured by SIAS level. The number of trials for each task is represented for each of the motions evaluated. In addition, the total number of trials for each group of patients is shown

### Data acquisition

Muscle activity was recorded using 18 wireless sEMG sensors (BTS FREEEMG; BTS Bioengineering Corp., Milan, Italy) symmetrically located on the following nine upper limb muscles (on both the left and right sides of the body): brachioradialis, pronator teres, biceps, triceps, anterior deltoid, posterior deltoid, pectoralis, infraspinatus, and elector spinae. These muscles were defined according to the guidelines of the Surface Electromyography for the Non-Invasive Assessment of Muscle Project [31]. Acquired data were digitalized using a sampling frequency of 1000 Hz and stored for subsequent processing.

### Basic sEMG processing

Raw sEMG data were high-pass filtered at 20 Hz to remove possible motion artifacts, rectified, and low-pass filtered at 32 Hz to preserve the key frequencies associated with muscle contractions. In both filtering stages, a fifth-order Butterworth filter was applied [32]. In addition, all channel amplitudes were standardized according to the median values recorded during the experimental session over the whole set of channels [33].

### Motion modeling

#### Definition

This model describes human motion as the contraction of muscles supported by a skeletal system that defines the degrees of freedom. Its main focus is the study of specific movements with clear starting and finishing points. From a detailed perspective, during the whole period of movement, each contributing muscle contracts one or more times (depending on the complexity of the motion) in synchrony with the whole set of contributing muscles [12, 34]. At the same time, to generate muscle contraction, a certain amount of electrical current must be applied for a certain amount of time over the muscle fibers [35, 36]. Under these conditions, a given movement can be associated with the amount of electrical power needed to generate the contraction of the participating muscles [37]. On the current work, electrical power will be quantized as the Root Means Squares (RMS) of the rectified sEMG. Total electrical power *P* is defined as the summation of the electrical power used by each muscle, *P*_*i*_, for *i*=1,2,3,...*N*, with *N* being the total number of muscles contributing to the movement (Eq. 1).

1 $$ P = P_{1} + P_{2} +... + P_{N}  $$

Figure 2a shows a graphical representation of Eq. 1. Moreover, each *P*_*i*_ value is redefined according to its proportional contribution to the total electrical power *P* through the use of *a*_*i*_ coefficients with *i*=1,2,3,...*N* (Eq. 2).

2 $$ P_{i} = a_{i}P  $$

Thus, *a*_*i*_ represents the percentage of *P* used by muscle *i*. Equation 3 is obtained by combining Eqs. 1 and 2 and provides normalized coefficients describing the division of total electrical power *P* among all the muscles contributing to the movement (Eq. 4). Hence, this analysis determines which muscles contribute most to the performance of a given movement.

3 $$ P = a_{1}P + a_{2}P +... + a_{N}P  $$

4 $$ 1 = a_{1} + a_{2} +... + a_{N}  $$

#### Approach applied during symmetric motions

Figure 2b shows a graphical representation of this approach applied during a symmetric motion. In equations 5–6, *P*_*r*_ and *P*_*l*_ represent the electrical power needed to perform the movement on the right and left sides of the body, respectively. Similarly, *a*_*i*_ and *b*_*i*_ are the coefficients representing the power associated with the muscles on each side of the body.

5 $$ P_{r} = a_{1}P_{r} + a_{2}P_{r} +... + a_{N}P_{r}  $$

6 $$ P_{l} = b_{1}P_{l} + b_{2}P_{l} +... + b_{N}P_{l}  $$

From this definition, it is possible to extract two parameters related to motor performance of symmetrical tasks. First is effective strength balance (ESB), which is defined as the comparison between *P*_*r*_ and *P*_*l*_ (Eq. 7). Changes in this parameter show differences among body sides regarding the effective muscle contractions emerging from decompensations in the absolute electrical power applied to them. Second is muscle coordination similarity (MCS), which is defined as the correlation coefficient between the values *a*_*i*_ and *b*_*i*_ extracted from the right and left sides, respectively (Eq. 8). This parameter compares the similarities between sides regarding how the total power is distributed among muscles (i.e., capturing the tendency towards muscle mirror symmetry) [38, 39].

7 $$ ESB = \frac{P_{r}-P_{l}}{P_{r}+P_{l}}\;\;\;\;\;\;\;\;\;\;\;\;\;\;\;\; -1\leq ESB \leq1  $$

8 $$ {\begin{aligned} MCS = \frac{n\sum_{n}^{1}a_{i}b_{i}-\sum_{n}^{1}a_{i}\sum_{n}^{1}b_{i}}{\sqrt{n\sum_{n}^{1}a_{i}^{2}-(\sum_{n}^{1}a_{i})^{2}} \sqrt{n\sum_{n}^{1}b_{i}^{2}-(\sum_{n}^{1}b_{i})^{2}}} \;\;\;\;\;\;\;\; -1\leq MCS \leq1 \end{aligned}}  $$

### Parameter extraction

After sEMG processing, the power per second of each muscle was quantized as the RMS of the rectified signal. In addition, the indexes defined in the motion model described in the previous section were computed. Coefficients *a*_*i*_ and *b*_*i*_ were extracted to show the benefits of their evaluation in assessing the importance of each muscle depending on the motion. Sets of *a*_*i*_ and *b*_*i*_ were represented as 18 values (associated with the nine muscles measured on each side of the body). In addition, the values of ESB and MCS were computed over the three groups of subjects to assess how different severity levels are affected by the two groups of neural adaptations that participate in motor performance.

## Results

### Muscle activity prints

**Fig. 3:**
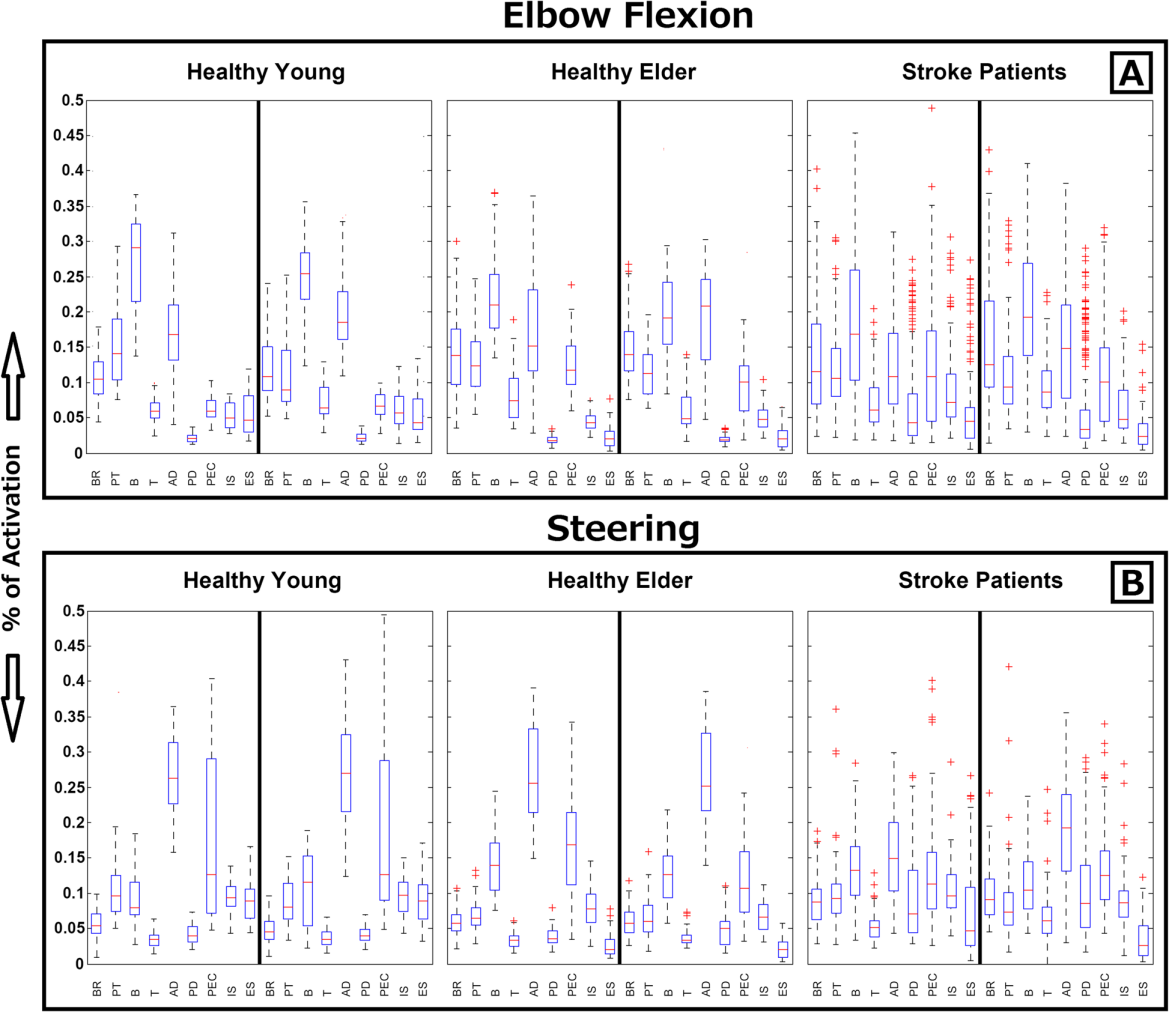
Muscle activity print. Muscle activity represented by the coefficients of dominant and non-dominant arms for the three groups of subjects: healthy young subjects (first column), healthy elder subjects (second column), and stroke patients (third column). The activity of each muscle is shown as a boxplot representing the 1.5 inter quartile ratio and average values (whiskers and red line) as well as the first and third quartiles of the distribution (top and bottom limits of the box) and outliers (red crosses). **a** Results associated with elbow flexion. **b** Results associated with the steering motion. BR, brachioradialis; PT, pronator teres; B, biceps; T, triceps; AD, anterior deltoid; PD, posterior deltoid; PEC, pectoralis; IS, infraspinatus; ES, elector spinae

The muscle activity print associated with each movement and subject group is summarized in Fig. 3 as a boxplot of *a*_*i*_ and *b*_*i*_. Figure 3a shows the muscle activity associated with elbow flexion, and Fig. 3b shows those associated with the steering motion. Moreover, each section is divided among three graphs (Fig. 3a1-3 and Fig. 3b1-3) representing the muscle activity print of healthy young, healthy elder, and post-stroke subjects, respectively. In this representation, coefficients on the right side of each graph corresponds to the muscles of the non-dominant arm of healthy subjects and the paretic arm of stroke patients. Therefore, coefficients on the left are used to represent the dominant arm of healthy subjects and non-paretic arm of stroke patients. The boxplot representation shows the distribution of these coefficients in terms of their 1.5 inter quartile ratio, and median values as well as the first and third quartiles of the distribution and outliers (as shown in the boxplot legend for Fig. 3).

The healthy young and elder muscle prints show how the important muscles involved in voluntarily motor control differ between the movements performed. During elbow flexion (Fig 3a), biceps and anterior deltoid muscles are dominant but strongly supported by brachioradialis, pronator teres, triceps, and pectoralis muscles. For the steering motion (Fig 3b), anterior deltoid and pectoralis muscles are the main carriers of the movement, while the remaining muscles contribute less power. This is expected as the dual steering system provides partial gravity support, which compensates for the power of supporting muscles used in the same task. Moreover, the rarity of outliers (red dots) among the healthy subjects indicates less variability on the sEMG data analyzed and therefore suggests the existence of stable muscle behaviors among healthy subjects associated with the specific motions evaluated. For stroke patients, even similar muscle trends can be observed on average (and for both motions), but the expected muscle prints are more difficult to distinguish. In addition, the number of outliers was drastically higher among patients, which is expected for the stroke population. Depending how the brain injury affects the neural processes controlling motion, each patient developed different control strategies manifested as muscle asymmetries. For example, in the case of elbow flexion, outliers demonstrate the tendency of some patients to use their posterior deltoid muscles to compensate for the lack of movement of the main muscles (i.e., anterior deltoid and biceps).

### Effective strength balance and muscle coordination similarity

**Fig. 4:**
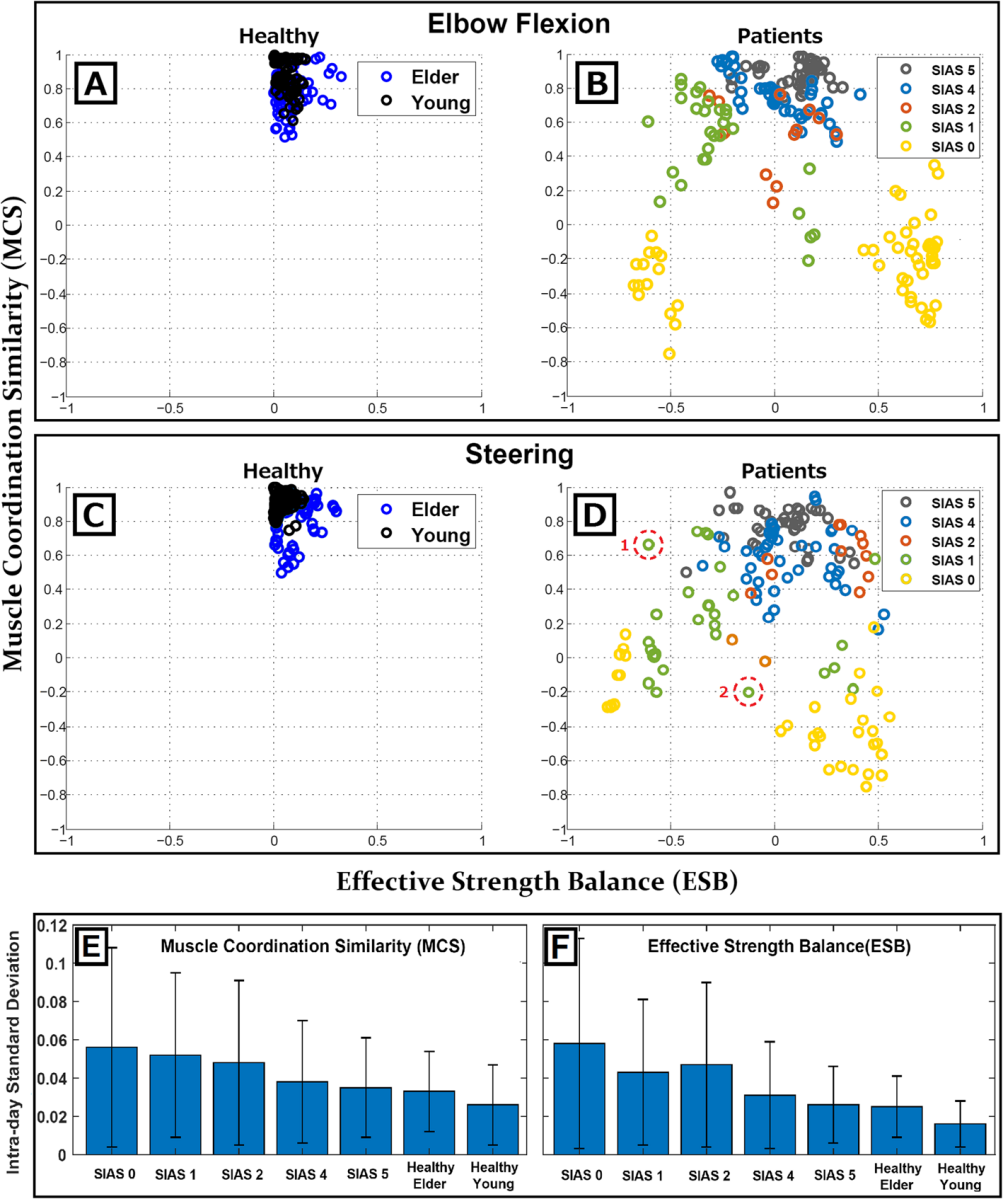
MCS–ESB representation. Graphs **a**-**d**: Bidimensional representation of motion performance according to the parameters MCS (*x*-axis) and ESB (*y*-Axis). Each row represents elbow flexion and steering motions, respectively. In the first column, healthy young and elder values are shown. In the second column, patient data are represented in different colors depending on the SIAS level. Graphs **e**-**f**: Intra-day variability of both parameters was measured from the standard deviation of the metrics obtained within single daily sessions. Values are represented for each group of subjects to show the average variability of MCS and ESB computed on consecutive trials. Values enclosed by red dotted circles on graph **d** represent examples of two patients whose motor impairment are classified at the same SIAS level even though their neurological origins are quite different

The scatter plots in Fig. 4 show the ESB values (*x*-axis) and MCS values (*y*-axis). Each point corresponds to the ESB and MCS values computed from a single experimental trial. In this representation, points with values close to *x*=0 are related to movements in which the left and right sides of the body require equal electrical power to contract the muscles. Depending on the level of dominance of one side of the body over the other, the value of the *x*-axis varies from -1 to 1, with -1 and 1 representing total dominance of the left and right sides, respectively. On the other hand, the *y*-axis shows the MCS values: *y*=1 represents the situation in which muscles are coordinated on the left and right sides of the body following the same strategy, that is, a situation of perfect muscle mirroring; *y*=0 implies a loss in the correlation of muscle activity between sides; and *y*<0 indicates the appearance of an asymmetrical correlation, which implies that symmetric muscles have switched roles, consisting of high activity on one side and poor activity on the other. This is a rare situation that must be considered to evaluate motion in severe stroke patients who are barely able or completely unable to move their paretic arms. Figure 4a–b show the results for parameters associated with elbow flexion while Fig. 4c–d represent those related to steering. The first graph for each motion (Fig. 4a and c) shows the data points corresponding to healthy young (black) and elder (blue) subjects. In the second graphs (Fig. 4b and d), the values obtained from stroke patients are represented by circles that differ in color according to their SIAS level. As all healthy subjects where right-handed, their ESB values were positive, in accord with the dominance of the right sides of their bodies. For stroke patients, the dominance of these parameters is directly associated with their paretic side (Table 1).

### SIAS versus MCS and ESB

**Fig. 5:**
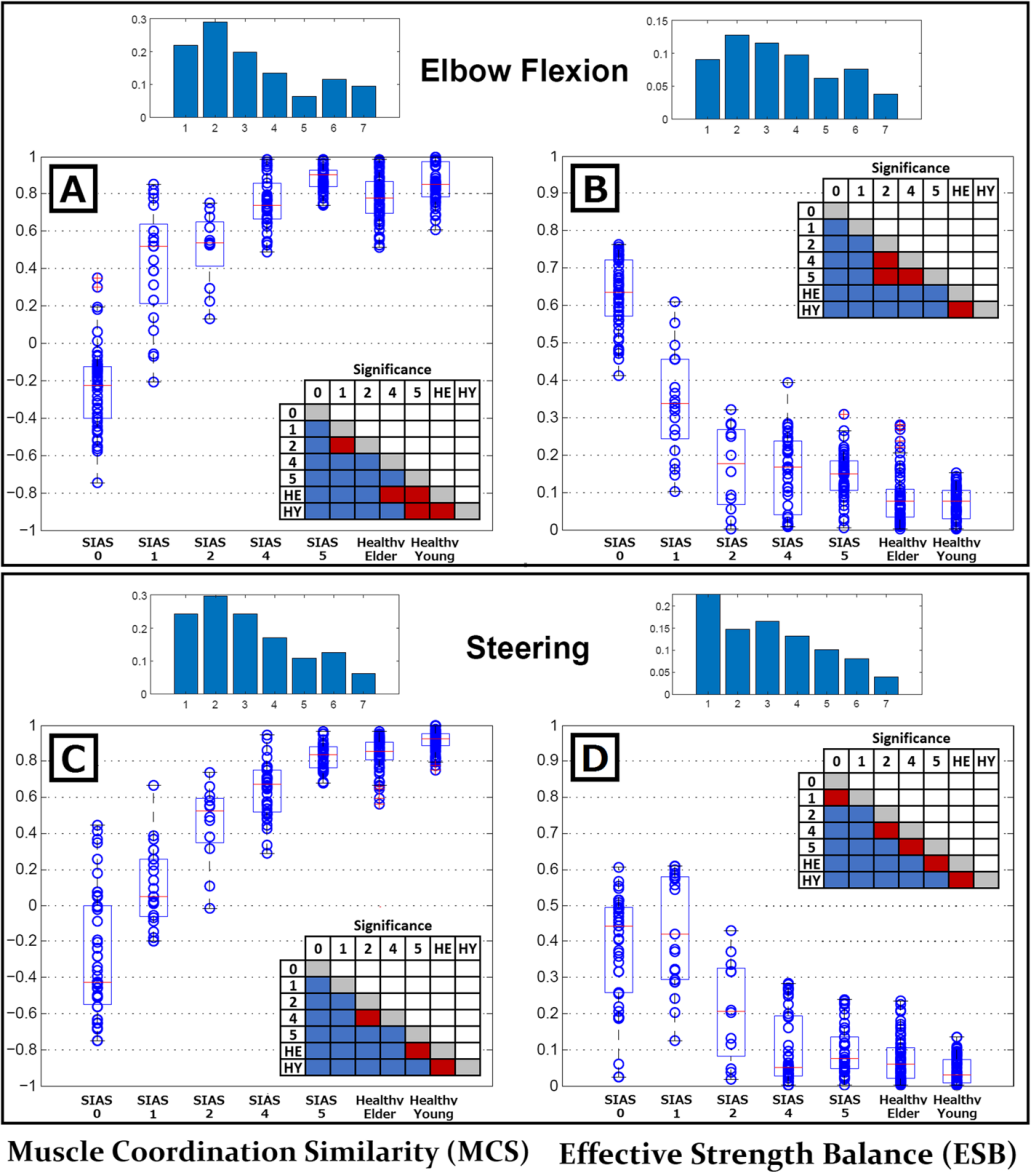
MCS and ESB versus SIAS level. In **a**–**d**, MCS and ESB values were compared among seven groups (with five SIAS levels from stroke patients, healthy elder subjects, and healthy young subjects). **a** and **b** show the parameters for the elbow flexion motion, while (**c**) and (**d**) show them for the steering motion. A Wilcoxon sum-rank test with a confidence interval of 95% was conducted to compare the healthy young group with the remaining groups and the healthy elder group with stroke patients. A Bonferroni–Holmes correction was applied to the confidence interval to control for the multiple comparisons of the presented analysis. Significance tables on each graph show in blue the pairs of results with significance differences and in red those without significance. Over each graph, the standard deviations measured from the set of data for each group was represented to show the evolution of these parameters according the severity of the injury

In Fig. 5, the *y*-axes represent MCS (A and C) and ESB (B and D) values for each SIAS level shown along the *x*-axes. Results are also represented in the form of boxplots showing the first and third quartiles as well as the 1.5 inter quartile ratio average, and outliers of each distribution. The results for healthy elder and healthy young subjects are also included to demonstrate the differences between these groups of subjects. The absolute value of ESB was used to assess these results in order to highlight how the SIAS level influences the side balance independently of the paretic arm. To evaluate the significance among groups, an all-vs-all statistical comparison was applied to each graph, namely, a Wilcoxon sum-rank test with a confidence interval of 95% [40] followed by a Bonferroni–Holms correction for multiple comparisons [41]. Multiple comparisons are represented in the form of tables that compare pairs of groups. Statistically significant pairs are coloured in blue while non-significant pairs are coloured in red. Furthermore, the standard deviation of each group was represented at the top of each graph to show the evolution of this parameter for each injury level. Results show, in general, significant differences between stroke and healthy groups. However, muscle coordination of SIAS 5 patients during elbow flexion show higher average value than healthy groups (although there is no statistical difference), suggesting better motion performance. This result might be related to the facts that elbow flexion was a task performed by these patients during their daily rehabilitation and SIAS 5 patients show, in many cases, motions undistinguish from healthy subjects.

## Discussion

It was initially argued that the mere evaluation of muscle coordination in patients recovering from strokes is insufficient to elucidate all neural processes involved in motor recovery. Even though the proper coordination of muscles is an important factor that contributes to the performance of an efficient motion, the ability to properly tune the effective strength of muscle contractions also plays an important role. By exploiting the symmetry of the human body, the model described in this work proposes two indexes directly related to the factors involved in motor recovery. The computation of these indexes for subjects ranging from healthy individuals to severe stroke patients provided interesting muscle performance results in terms of the factors involved in motor recovery.

For the healthy young and elder subjects, there were clear similarities between body sides both in muscle coordination (MCS) and muscle effective strength (ESB) (Fig. 4a-c). Also, the statistical comparison performed between these two groups on Fig. 5 shows no significant differences, suggesting the absence of age dependence on these factors during simple symmetrical motions. The ability to efficiently coordinate muscles and tune their contraction strength creates a perfect scenario for the study of muscle synergies. Healthy subjects are able to maintain stable synergies throughout a range of variation within a task by increasing or decreasing motor unit recruitment, thereby tuning the strength of individual muscles.

However, in the case of stroke survivors, the ability to coordinate muscles and tune their effective contraction strength are affected differently depending on the area and size of the lesion. This is clearly represented by MCS and ESB indexes in Fig. 4b–d. Even within patients classified with the same paralysis level, their performance regarding these two factors hugely differs. The comparative example highlighted in Fig. 4d shows how a patient with almost healthy muscle coordination (dashed-circle 1) is classified as SIAS 1 owing to their inability to balance strength among sides of the body. At the same time, another patient, also classified as SIAS 1, shows no problems balancing the strength among sides but completely lacks the ability to coordinate their muscles properly (dashed-circle 2). Moreover, by evaluating the standard deviations shown in Fig. 5, it can be seen that both MCS and EBS present increasing dispersion for patients with lower SIAS levels. This suggests that muscle coordination and muscle strength tuning are less coupled in severe patients, contributing to an explanation of the appearance of a wide range of synergistic abnormalities when evaluating them.

Accordingly, the application of models that explain motor control based on neural processes in charge of motor coordination is incomplete in studying motor recovery. In this scenario, other neural processes often related to recovery of motion strength also play an important role. The identification of the neurological origins of post-stroke motor impairment is a relevant factor for the assignment of effective rehabilitation therapies. In that regard, as supported by the data analyzed, it appears that the model described and the indexes inferred from it have the potential to distinguish among the different factors affecting motor recovery based on the analysis of sEMG signals. In addition, the reduced setup and simplicity of calculating these indexes make them easy tools for standardizing rehabilitation therapies.

## Conclusions

This research focused on the development of a model for extracting information from sEMG signals to quantify the neural mechanisms behind motor recovery. Given the physiological properties of sEMG signals and current knowledge about the neural processes in charge of recovering motor strength [22, 23, 23], motion performance was defined as the combination of two main factors: (1) coordination of muscles (representing the synergetic behaviors observed in healthy subjects) and (2) tuning of strength through individual muscle contractions (representing the neural processes underlying recruiting motor units and strengthening neural synaptic and structural connections). The model-inferred indexes MCS and ESB, which represent these factors, were validated using sEMG data recorded from three different groups of subjects during two different motions. Even though these factors seemed closely related during healthy subject motion, results obtained from stroke patients suggest that brain damage contributes to decoupling both factors, thereby creating a range of motor impairments that are commensurate with stroke severity. Current standard stroke rehabilitation therapies are focussed on the improvement of muscle coordination [42, 43], however our results show that some patients with poor motor performance show good muscle coordination (MCS). This fact suggest that a direct use of the metrics developed could be used to choose between a muscle coordination or muscle strength oriented therapy.

Finally, it is important to note that the present method is based on a comparison between paretic and non-paretic body sides. Even though this is a commonly accepted methodology in stroke research, it assumes a healthy condition of the non-paretic side. To avoid the misinterpretation of results, researchers using these parameters should confirm the condition of the non-paretic area used as the empirical expectation. However, the current work is not intended to study the rehabilitation benefits of the specific motions evaluated. The recorded sEMG data were used only to validate the model proposed and show its potential as a tool to evaluate motor recovery. The model consistency should be also further analyzed by including a wider range of tasks to those evaluated on this work. It should be also mention that the methodology proposed do not intend to provide ultimate solutions to all current challenges in the field of motor rehabilitation. For instance, many daily tasks are unimanual or require independent actions on each limb. The system proposed, as happens also with many other rehabilitation techniques, cannot be used on a daily environment. However, it allows the use of the non-paretic areas of the body, within a controlled clinical environment, in order to potentiate the recovery of the paretic areas by exploiting the symmetrical structure of human body. In fact, the recovery of specific motions on the paretic limb using feedback from the non-paretic side is a common technique on rehabilitation [44]. Nevertheless, the use of our model for unimanual task could be discussed for future studies focused on increasing patients’ motion dexterity.

This work is the beginning of a wider research program that is intended to increase the current understanding of motor recovery in order to improve rehabilitation therapies. Accordingly, the next research stages include the following objectives: (1) development of a system that provides real-time feedback about ESB and MCS to patients during rehabilitation; (2) integration of ESB and MCS with the study of muscle synergy development during rehabilitation to better understand the neural processes following recovery; and (3) development of a rehabilitation protocol adaptable to specific patient conditions and neural recovery strategies based on the results obtained in the this and future studies.

## Data Availability

sEMG data from healthy subjects and stroke patients could be requested by email to the authors.

## Abbreviations

ESB: Effective strength balance
MCS: Muscle coordination similarity
RMS: Root mean squares
sEMG: Surface electromyography
SIAS: Stroke impairment assessment set
